# Optimisation and Use of Humanised RBL NF-AT-GFP and NF-AT-DsRed Reporter Cell Lines Suitable for High-Throughput Scale Detection of Allergic Sensitisation in Array Format and Identification of the ECM–Integrin Interaction as Critical Factor

**DOI:** 10.1007/s12033-013-9689-x

**Published:** 2013-07-27

**Authors:** Xiaowei Wang, Paul Cato, Hsiu-Chen Lin, Tongen Li, Daniel Wan, Marcos J. C. Alcocer, Franco H. Falcone

**Affiliations:** 1 School of Biosciences, Sutton Bonington Campus, Loughborough, LE12 5RD UK; 2 Division of Molecular and Cellular Science, School of Pharmacy, University of Nottingham, Science Road, Nottingham, NG7 2RD UK

**Keywords:** RBL, Basophil array, Adhesion, Fibronectin, VLA-4, Reporter system, GFP, DsRed

## Abstract

We have previously described a microarray platform combining live basophils with protein arrays suitable for high-throughput detection of sensitisation against allergens. During optimisation of this technique, we observed severe losses of adhering cells during the washing steps, particularly after activation. In order to preserve cell binding, we tested the cell adhesion characteristics of different extracellular matrix proteins: human collagen I, fibronectin (FN) from bovine plasma and laminin (LN). FN was more effective than LN and collagen. Cell detachment after activation was in part due to reduced surface expression of VLA-4, the main ligand for FN, which was significantly decreased within 15 min of stimulation with 1 μg/mL calcium ionophore A23187, reaching a minimum after 2 h then slowly recovering. These optimised conditions were used for testing of well-characterised sera from allergic patients using two newly developed rat basophil leukaemia stable reporter cell lines (RBL NF-AT/GFP and RBL NF-AT/DsRed), which both express the human high-affinity IgE receptor alpha chain (FcεRIα). Both cell lines were able to detect sensitisation to specific allergens showing the expected bell-shaped dose–response curve, and correlated (*R* ^2^ = 0.75) with the standard beta-hexosaminidase assay, which is not suitable for an array format.

## Introduction

Basophils and mast cells play a central role in allergic reactions [1]. Crosslinking of Immunoglobulin E (IgE) bound to cell surface high-affinity IgE receptor (FcεRI) with antigen triggers intracellular signalling events, leading to multiple cellular responses involving cell degranulation and release of various chemical mediators such as histamine, chemokines, lipid mediators, and cytokines that cause inflammation and common allergic symptoms [2].

Laboratory diagnosis of allergy is usually performed by measuring levels of allergen-specific IgE (sIgE) in patients’ serum. Traditionally each suspected allergen is tested individually, requiring larger amounts of serum and increasing the overall cost. More recently, technological advances have enabled the simultaneous assessment of specific IgE levels for up to a hundred of allergens, either using multiplex flow cytometry [3] or allergen arrays [4–6].

While these new technologies in combination with recombinant or purified allergens allow component resolved diagnosis of allergic sensitisation [7], there are still unsolved issues regarding the clinical relevance of specific IgE measurements, as the existence of specific IgE binding does not always correlate with clinical symptoms. False-positive results are obtained e.g. due to cross-reactivities with pan-allergens or IgE directed against cross-reactive carbohydrate determinants, which may not have the ability to engage surface-bound IgE productively on mast cells and basophils, and are thus often clinically irrelevant [8, 9].

A recent study found that the concordance between sIgE measurements and skin prick tests (SPT) in allergy to cow’s milk and hen’s egg is unexpectedly low [10]. Similar inconsistencies between tests have also been reported for allergy to hymenoptera venom [11]. As a result, diagnosis of Type 1 Allergy is still best performed using a combination of sIgE, SPT, clinical history, and, in the case of food allergy, oral challenge tests [12].

Thus there is clearly a need for more clinically relevant diagnostic methods, while taking advantage of component resolved diagnosis and the medium- to high-throughput capacity of novel technologies. In a previous study, we have demonstrated that humanised rat basophil leukaemia (RBL) cell lines could be sensitised by human IgE or sera from allergic patients and degranulation monitored in a cell protein microarray system containing anti-IgE or matching allergen [13]. During the optimisation of the procedure, we observed that in the absence of fixation, sensitised basophils detached from the array surface during washing after activation. The degree of detachment strongly depended on the type of stimulation used, whether proteins were used for coating and the washing protocol.

Although receptor crosslinking is central to the degranulation cascade, other processes potentiate the cell activation process. The adhesion of mast cells and basophils to extracellular matrix (ECM) proteins such as fibronectin (FN), collagen (CO), laminin (LN) or vitronectin (VN) for instance has been shown to play an important role in enhancing degranulation of rat basophil leukaemia RBL-2H3 cells [14–16]. RBL cells do not adhere to uncoated surfaces or show any adherence in the absence of Ca^2+^ [14]. RBL adherence to ECM proteins is mediated by specific cell surface receptors that belong to the integrin family [16].

Many integrins bind the tripeptide Arg-Gly-Asp (RGD) sequence present on ECM proteins, originally described on FN [17]. The β1 integrin subfamily in particular includes receptors that bind to the ECM proteins FN, CO and LN, and have been designed VLA (very late antigen) [18]. VLA-4 and VLA-5 have been shown to mediate RBL-2H3 cell adhesion to FN [16]. VLA-4 recognises the CS-1 sequence present in the type III connecting segment (IIICS) of the cell-binding domain of FN, while VLA-5 recognises the RGD sequences [16, 19].

Our long-term aim is to develop a fully automated basophil array; in order to achieve this, two major obstacles had to be overcome: the cell detachment during the washing steps and the development of reporter systems suitable for an array format. While a NF-AT–luciferase reporter system has been recently developed by Nakamura et al. [20] for diagnostic purposes, luciferase-based systems require cell lysis before measurement of luciferase activity. However, a lysis step will disconnect the signal from its exact position on the array and is, therefore, not suitable in this format. In this work, we describe optimised conditions for the use of newly generated reporter systems which are suitable for use in high-throughput formats such as allergen arrays, providing a biological readout.

## Materials and Methods

### Cells and Cell Culture

All experiments were performed with rat basophilic leukaemia cell line (RBL-703/21) transfected with the human FcεRI receptor α chain generated at Paul-Ehrlich-Institut (Langen, Germany; kindly donated by Stefan Vieths and Lothar Vogel). Cells were grown in MR 80/20 medium: 80 % (v/v) Minimal Essential Medium (MEM; GIBCO) plus 20 % (v/v) RPMI-1640 medium (Sigma-Aldrich,UK) supplemented with 5 % (v/v) heat-inactivated Foetal bovine serum (FBS; GIBCO, UK), 2 mM l-glutamine, 50 IU/mL penicillin and 50 mg/mL streptomycin (Invitrogen, Paisley, UK). The transfected cells were cultured in the presence of 1 mg/mL geneticin sulphate (G-418; Sigma-Aldrich) at 37 °C in a humidified atmosphere containing 5 % CO_2_. Recombinant Allergens were from Greer Labs (Canada).

### Binding of Cells to Array Slides

Polyclonal goat anti-human IgE 0.5 mg/mL (Biosource, Camarillo, CA, USA) was hand printed (~0.1 μL/spot) onto 16-Pad FAST slides (Whatman Schleicher & Schuell, Dassel, Germany). The diameter of each spot was approximately 450 μm. The slides were then blocked with 3 % (w/v) BSA in PBS for 30 min at 37 °C, dried, and stored until use at room temperature. Before incubation with the cells, slender strips of adhesive tape (Gene-Frame, ABgene, Epsom, UK) were attached to margins of the coated slide surface to produce a rectangular incubation area for cell/protein interaction. The IgE- or serum-sensitised rat basophilic cell lines were harvested and re-suspended in 0.25 % (w/v) BSA in Dulbecco’s PBS (DPBS) (Lonza, Belgium) at a density of 2.4 × 10^4^ basophils/100 μL and transferred onto the 16-Pad FAST slide. Cells were incubated for 2 h at 37 °C in a humidified atmosphere with 5 % CO_2_. The slides were gently swirled manually for 10 s every 30 min. After incubation, unbound cells were washed off the slide by loading onto a slide rack and washing in a reservoir containing PBS at room temperature with gentle immersion and lifting for 5 s twice manually, followed by fixation for at least 1 h in PBS containing 1 % (v/v) formaldehyde. Bound cells on FAST slides were observed to be wet using a conventional light microscope with a 4× objective lens. Images were captured and quantified using Image Pro Plus software (Media Cybernetics, Bethesda, MD, USA) equipped with a Nikon Micropublisher 3.3 RTV Labophot-2 camera (Qimaging, Surrey, Canada) and were processed using Adobe Photoshop software.

### Surface Expression of Human FcεRI Receptor

In order to optimise culture conditions leading to the surface expression of the human high-affinity IgE receptor, RBL-703/21 cells were incubated with increasing concentrations of IgE for different lengths of time. Normal rat serum was heat-treated (55 °C, 30 min) to inactivate complement and added to cells in medium at 10 % (v/v) in order to reduce potential background staining caused by non-specific antibody binding. Surface expression of FcεRIα was detected by mouse IgG2b CRA-1-FITC monoclonal antibody (CosmoBio, Tokyo, Japan) which is specific for the α-chain of human FcεRI and does not compete for IgE binding. FITC-labelled mouse IgG2b monoclonal antibody specific for keyhole limpet haemocyanin (KLH) was used as the isotype control (R&D Systems, UK). FITC-labelled antibodies (10 μg/mL) were added to 1 × 10^5^ RBL-703/21 in medium and incubated at 4 °C for 30 min. These steps were performed under aseptic conditions in the dark. Samples were then spun at 250 g for 5 min and washed in DPBS (without Ca^2+^/Mg^2+^) twice to remove unbound antibody. Finally, cells were fixed in 0.5 % (v/v) formaldehyde in DPBS and analysed in a FACScan flow cytometer (Beckman-Coulter, USA).

### Adhesion Assay

FN from bovine plasma (Sigma-Aldrich, UK) was coated at concentrations ranging from 0.625 to 20 μg/mL in a Maxi-Sorp 96-well Elisa plate (Nunc, UK), with one 0.5 % (w/v) bovine serum albumin (Millipore, UK) coated (negative control) and one uncoated well (blank control) at 4 °C overnight prior the assay. 1 × 10^6^ cells/mL of RBL-703/21 were sensitised with human IgE (1 μg/mL) (AbD Serotec, UK) o/n at 37 °C/5 %CO_2_. After 16 h, untreated and IgE-sensitised cells were re-suspended in serum-free medium to a final concentration of 1 × 10^6^ cells/mL. The pre-coated ECM plate was blocked with 0.5 % (w/v) BSA (Millipore, UK) at room temperature for 1 h. One group of the normal cells and the human IgE-sensitised cells were induced with calcium ionophore A23187 (1 μg/mL) (Sigma-Aldrich, UK) and anti-human IgE (2 μg/mL) (Vector Labs, UK) separately, cell suspension (100 μL) were immediately added to each well of the plate, each condition in triplicates, and incubated for 2 h at 37 °C/5 %CO_2_. After incubation, the unattached cells were removed by washing by aspiration and replacing with fresh MR80/20 medium three times. The bound cells were stained with 0.1 % (m/v) crystal violet (Millipore, UK) for 10 min, lysed with 10 % (v/v) acetic acid (Fisher, UK) and absorbance was read by a microplate reader (Model 680 XR, Bio-Rad, USA) at 550 nm.

### Immunofluorescence Flow Cytometry

The surface expression of VLA-4 integrin was analysed by immunofluorescence flow cytometry. Untreated and IgE-sensitised cells were harvested and resuspended as described above. After washing, cells were incubated in the absence or presence of calcium ionophore A23187 (1 μg/mL) (Sigma-Aldrich, UK) or polyclonal goat anti-human IgE (2 μg/mL) (Vector Labs, UK) at 37 °C/5 %CO_2_ for 0, 5, 30, 60, 120, 180 and 240 min, then incubated with 2.5 μg/mL fluorescein isothiocyanate (FITC)-conjugated anti-human VLA-4 integrin antibody (Novus, UK) at 37 °C for 1 h. After two washes, cells were fixed with 0.5 % (v/v) formaldehyde (Sigma-Aldrich, UK) on ice, and analysed in a FACScan flow cytometer (Beckman-Coulter, USA). Data were processed and analysed using Weasel software (WEHI, Australia).

### Construction of Reporter Cell Lines

The forward primer 5′-ATAATTAAAGATCTGCAGAACTGTGAATGCGCAAACC-3′ and reverse primer 5′-ATAATTAATGAATTCGAGCTCGGTACCCGG-3′ were used to amplify the NF-AT enhancer from pNF-AT-hrGFP plasmid (Stratagene, US), with overhangs and BglII and EcoRI restriction sites, respectively (underlined). The forward primer 5′-ATATAATAGAATTCACCATGGATAGCACTGAGAACGTCATCAAG-3′ and reverse primer 5′-ATATTTAATCTCGAGCTACTGGAACAGGTGGTGGCGG-3′ were used to amplify the DsRed-Express2 gene from pDsRed-Express2-1 plasmid (Clontech, US) with overhangs and EcoRI and XhoI restriction sites, respectively (underlined). Cycling conditions used were: Initial denaturation 94 °C 600 s, followed by 35 cycles of denaturing (94 °C, 60 s), annealing (61 °C, 60 s) and extension (72 °C, 60 s) with a final extension (72 °C, 600 s), on a GeneAMP PCR system 9700 (Applied Biosystems, USA). PCRs were performed using AmpliTaq Gold DNA Polymerase (Invitrogen, UK). To construct the NF-AT/DsRed plasmid, the NF-AT enhancer and DsRed-Express2 PCR products were sequentially ligated into pUB6/V5-His A (Invitrogen, UK) plasmid using T4 DNA ligase (Promega, UK) after digestion of vector with appropriate restriction enzymes as indicated above. DNA sequences were verified by DNA sequencing (Sigma-Aldrich, UK). pNF-AT-hrGFP and NF-AT/DsRed plasmids were amplified in DH5α *E. coli* and purified using a Plasmid Maxi Kit (QIAGEN, Germany) using standard molecular biology protocols and following the manufacturer’s supplied instructions, respectively. Plasmid purity was assessed by OD260/280 on a Nanodrop 1000. Each plasmid was transfected into RBL-703/21 cells by electroporation. In a 4-mm electroporation cuvette (Bio-Rad, US), 15 μg plasmid was added to 4 × 10^6^ cells in 100 μL MR80/20 medium. Electroporation was carried out at 250 V, 250 μF in a GenePulser Xcell (Bio-Rad, UK). Cells were transferred from the cuvette into 6-well plates containing cell culture medium. 3 days after transfection, stable transfectants were selected using 1 mg/mL hygromycin B (Invitrogen, UK) for pNF-AT-hrGFP transfectants and 20 μg/mL blasticidin S (Invitrogen, UK) for NF-AT/DsRed transfectants. After 6-week selection by hygromycin B, or 1-week selection using blasticidin S, cells were activated using 1 μg/mL human IgE (AbD Serotec, UK) and 2 μg/mL anti-human IgE (Vector Laboratories, UK), and single fluorescent cells were sorted into each well of 96-well plates using a MoFlo cell sorter (Beckman Coulter, USA). The most responsive clones, as indicated by the highest fluorescence after activation, were chosen and maintained in 1 mg/mL hygromycin B or 20 μg/mL blasticidin S.

### Reporter Assay

Reporter gene activation was measured in 384-well plates due to the unavailability of suitable instrumentation with the ability to image array-bound fluorescent cells in a wet environment at sufficiently high resolution (see “Discussion”). The wells were pre-coated with 20 μg/mL FN at 4 °C overnight. Cells were then seeded at a density of 50,000 cells/well and sensitised overnight with 1:20 or 1:40 diluted serum of allergic patients or monoclonal myeloma IgE (1 μg/mL) in 30 μL of MR 80/20 medium. The next day, the medium was replaced with fresh cell culture medium to remove unbound IgE before adding recombinant Bet v1, Phl p1 (both from Biomay AG, Vienna, Austria), Par j2 allergen (Bial Industrial Farmacéutica S.A, Bilbao, Spain) or 2 μg/mL polyclonal anti-human IgE antibody (Vectorlabs, Peterborough, UK) as positive control for induction. Activation of reporter cell lines was measured after 20 h using a Typhoon Trio Scanner (Amersham Bioscience, Sweden) at Ex:Em: 488/520 nm for NF-AT-hrGFP-transfected cells, and Ex/Em: 532/580 nm, for NF-AT-DsRed-transfected cells. Fluorescence intensity in each well was quantified and analysed by Image Quant software (Gel Life Science, USA). Fluorescent images were taken with an EVOS *fl* microscope (Advanced Microscopy Group, Mill Creek, Washington) at the indicated magnification.

### Statistical Analysis

Statistical analysis was performed using a two-tailed Student’s *t* test. *p* values <0.05 were considered statistically significant (*p < 0.05; **p < 0.01, or ***p < 0.001). Multiple comparisons were performed with ANOVA and post-hoc Tukey or Bonferroni tests using IBM SPSS Statistics Version 16 (IBM, NY, USA).

## Results

### Effect of IgE Sensitisation on FcεRIα Chain Expression and Array Binding

**Fig. 1:**
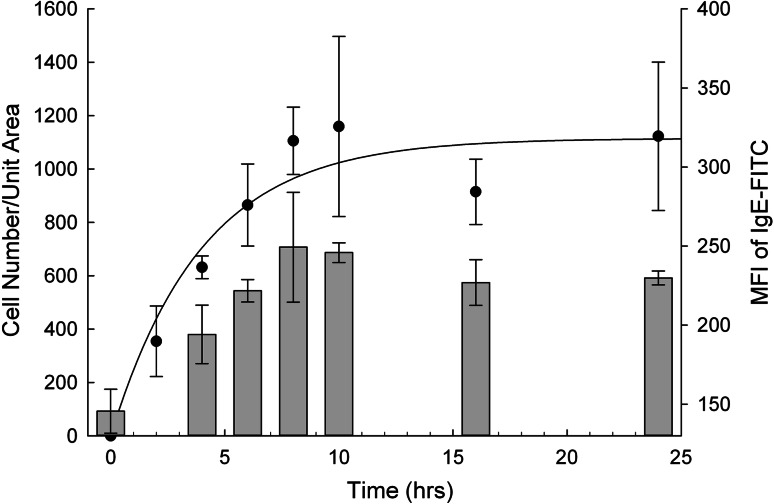
Time course of RBL-703/21 binding to IgE-coated microarray per unit area. RBL-703/21 cells were sensitised with 10 μg/mL monoclonal human IgE-FITC for different lengths of time and captured on FAST slides coated with polyclonal anti-human IgE. After washing and fixation, images were taken and the number of cells captured was determined by image analysis (*left axis*, *full circles*). Surface-bound IgE was measured by flow cytometry and expressed as Median Fluorescence intensity (*right axis*, *bars*). Data are expressed as mean ± SD of three separate experiments

A time course experiment was set up to determine the optimal incubation time for the sensitisation of the humanised cell line. In this experiment the RBL-703/21 cell line was incubated with 10 μg/mL monoclonal human IgE for various lengths of time. After incubation, sensitised cell lines were harvested and bound to array slides as described in Methods. Polyclonal anti-human IgE was used for capture in these experiments. Flow cytometric analysis of FcεRI receptor demonstrated (Fig. 1, bars) that longer IgE sensitisation times resulted in higher surface expression of FcεRIα chains, reaching a plateau after 8–10 h. Longer IgE incubation time also resulted in a larger number of cells immobilised to the membrane array displaying similar kinetics. Sensitisation time in excess of 10 h did not increase surface expression of FcεRI α chains and did not increase the density of cells bound to the anti-IgE spots on the array (dark circles in Fig. 1).

### Cell Density Experiment

**Fig. 2:**
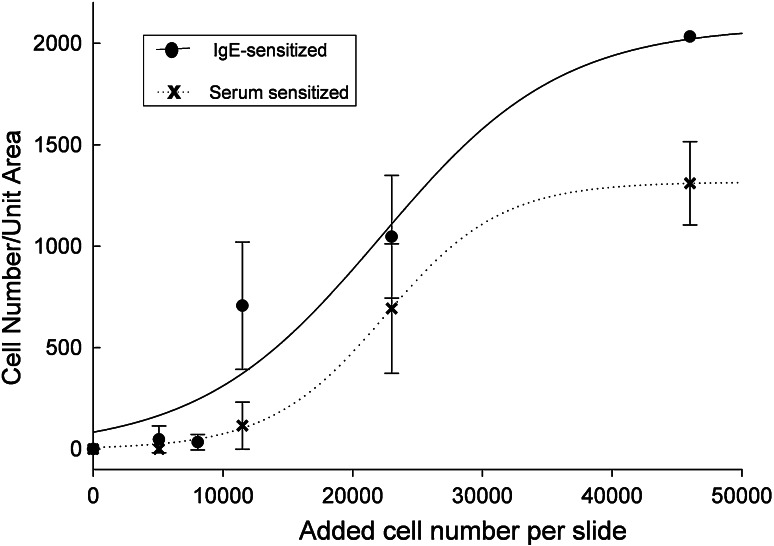
Binding of cells per unit area in dependence of initial cell density added to the array slides. RBL-703/21 cells were sensitised overnight with 2 % human serum (*crosses*, *dotted line*) or myeloma IgE (*black circles*, *continuous line*) and captured with polyclonal IgE printed on FAST slides. Varying cell densities were incubated with the arrays and the total amount of cells bound evaluated as described in Methods. Data are shown as mean ± SD of three separate experiments. Sigmoidal best fit curves for both conditions are shown

Cell density experiments were carried out to determine the optimal cell number for cell binding to FAST slides. In these experiments, RBL-703/21 cells were sensitised with 2 % patient serum or 10 μg/mL monoclonal human IgE for 16 h at 37 °C in humidified air containing 5 % CO_2_. Sensitised cell lines were then harvested and different amounts of cells were incubated with the array pads following the standard binding protocol. Again polyclonal anti-human IgE was used for capture in these experiments. As shown in Fig. 2, increasing cell densities applied to the array resulted in higher densities of bound cells, as expected. Sensitisation with 2 % patient serum was less efficient than myeloma IgE, but still resulted in dose-dependent cell adhesion to the array. The stronger attachment using myeloma IgE was probably due to the higher IgE concentration used compared with serum, allowing complete occupancy and/or upregulation of high-affinity IgE receptor on the cells, thus stronger interaction with the polyclonal antibody used for cell binding to the FAST slide.

### Inclusion of FN Increases Adhesion

**Fig. 3:**
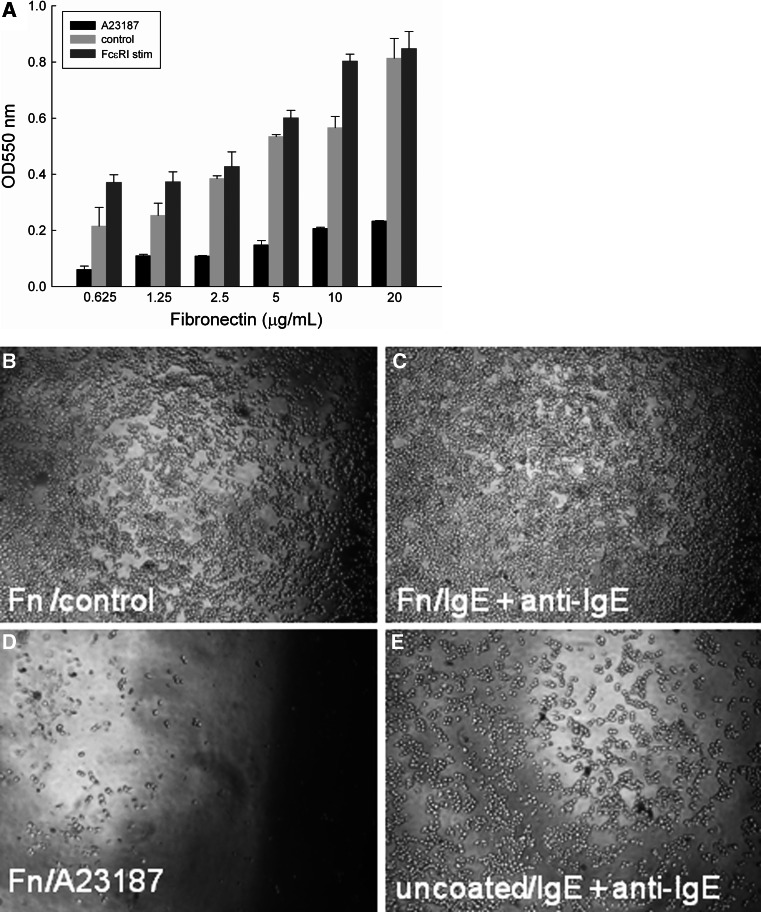
**a** Attachment of RBL-703/21 cells to FN. Different concentrations of FN (0.625–20 μg/mL) were used for coating, cells were activated with 1 μg/mL A23187, 2 μg/mL anti-IgE, or left untreated (control). 120 min after activation, cells were washed three times and the amount of adherent cells was quantified by a colorimetric assay using crystal violet. Data expressed are mean ± SD from triplicate determinations. Representative of three independent experiments with comparable results. Multiple comparison for two variables was obtained with a two-way ANOVA with Bonferroni post-hoc test to assess the statistical significance of FN and the type of stimulation for cell attachment/detachment. There was a highly significant effect of FN (*F*(5,32) = 163.458, *p* < 0.001) and the type of stimulation (*F*(2,36) = 611.867, *p* < 0.001) on cell attachment. **b**–**e** ×50 Light microscopy magnification of RBL 703-21 cells, after 2-h binding to 20 μg/mL FN without stimulation (**b**), activation with 2 μg/mL anti-IgE with FN (**c**), or activation with 1 μg/mL A23187 with FN (**d**), or activated with 2 μg/mL anti-IgE without FN coating (**e**)

IgE-sensitised RBL-703/21 cells bound to FN in a dose-dependent manner (Fig. 3a), with the highest binding at a concentration of 20 μg/mL. Activation with anti-human IgE antibody enhanced cell adhesion compared with IgE alone (control, Fig. 3b, c) at suboptimal FN concentrations (0.625–10 μg/mL, Fig. 3a). Addition of calcium ionophore A23187 caused marked cell detachment from FN within the 2-h incubation period (Fig. 3d), even with the highest used FN concentration. Detachment of cells after activation in the absence of FN was also seen in anti-IgE-stimulated cells (Fig. 3e), albeit less pronounced than with the calcium ionophore.

### VLA-4 is Strongly Downregulated by A23187 but not by IgE-Dependent Activation

**Fig. 4:**
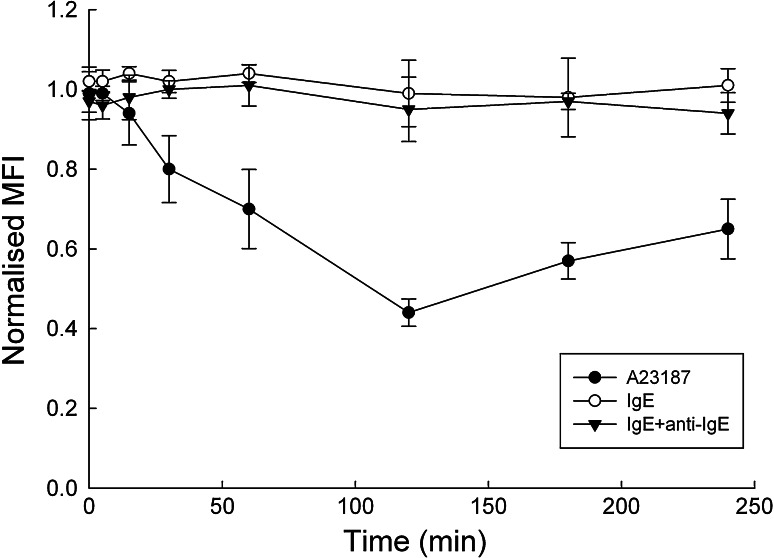
Time course of VLA-4 Integrin surface expression on RBL-703/21 cells stimulated with anti-IgE or 1 μg/mL A23187. Data are expressed as mean ± SD from three independent experiments. MFI values were normalised to 100 % for the unstimulated, unsensitised cells within each experiment. One-way ANOVA was used to determine statistical significance in changes of surface levels of VLA-4 after stimulation. Stimulation with A23187 led to highly significant downregulation of integrin expression (*F*(7,16) = 30.074, *p* < 0.001), starting at 30 min, as shown by Tukey post-hoc comparison. In contrast, neither unstimulated (*F*(7,16) = 0.561, *p* = 0.767), nor anti-IgE-stimulated cells (*F*(7,16) = 0.775, *p* = 0.617) showed any significant VLA-4 surface expression changes

Because of the strong detachment observed after A23187 stimulation even in the presence of 20 μg/mL FN as coating agent, we assessed the surface expression of VLA-4, the main FN receptor on RBL cells [16]. As shown in Fig. 4, flow cytometric analysis of surface VLA-4 expression clearly demonstrated a fast downregulation of the integrin already measurable 15 min after addition of A23187, reaching a maximum after 2-h incubation, then recovering steadily in the next 2 h. In contrast, IgE-dependent activation did not result in measurable changes in surface VLA-4 levels, and was very similar to sensitised, unstimulated cells.

The strong, progressive downregulation of VLA-4 after A23187, but not anti-IgE stimulation, may explain the stronger and almost complete detachment of cells seen with ionophore stimulation. To the best of our knowledge, this is the first report of a differential VLA-4 integrin downregulation on RBL cells upon receptor-mediated versus non-receptor-mediated stimulation.

### Differential Effects of FN, CO and LA on Cell Attachment After Activation

**Fig. 5:**
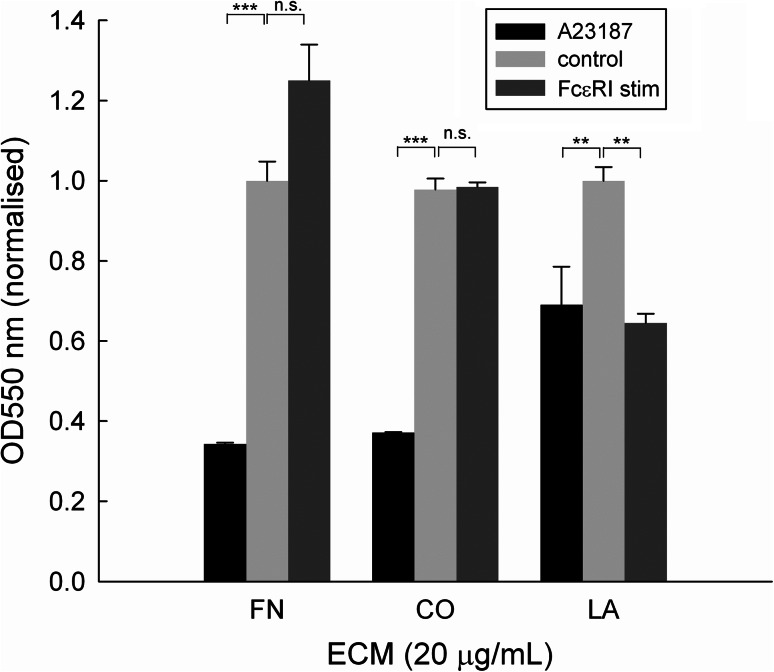
Attachment of RBL-703/21 cells to FN, type I CO or LA. 20 μg/mL of each ECM protein were used for coating; cells were activated with 1 μg/mL A23187, via crosslinking of FcεRI-bound IgE, or left untreated (control). 120 min after activation, cells were washed and the amount of adherent cells quantified by a colorimetric assay using crystal violet. Data expressed are mean ± SD from triplicate determinations. Asterisks show results of unpaired *t* test; *n.s.* not significant, **p < 0.01, ***p < 0.001. Data were normalised for OD550 nm of control unstimulated cells

Next we assessed the effect of different ECM proteins on RBL-703/21 attachment during activation. All three ECM were used at 20 μg/mL, which had shown the highest cell-binding capability for FN. As shown in Fig. 5, receptor-independent stimulation with A23187 led to more than 60 % cell detachment during washes in the presence of FN or type I CO but only approximately 25 % with LA. FcεRI-dependent stimulation appeared to lead to increased binding for FN, although this did not achieve statistical significance, but not for CO and LA. In the latter case, ionophore or FcεRI-dependent resulted in a similar loss of cells after activation despite the presence of LA; however, cell detachment was less pronounced than with FN or CO. Furthermore, we also compared human with bovine FN but saw no difference between the two (data not shown).

### NF-AT-GFP and NF-AT-DsRed are Induced After Allergen-Dependent IgE Crosslinking

Based on the results shown above, all subsequent experiments were performed in the presence of 20 μg/mL FN. Our next aim was to use these optimised conditions with suitable RBL reporter cell lines, as the array format requires that the activation marker used for assessment of activation remains confined to the cells, in contrast to soluble mediators such as histamine or beta-hexosaminidase, which are released by activated RBL cells.

**Fig. 6:**
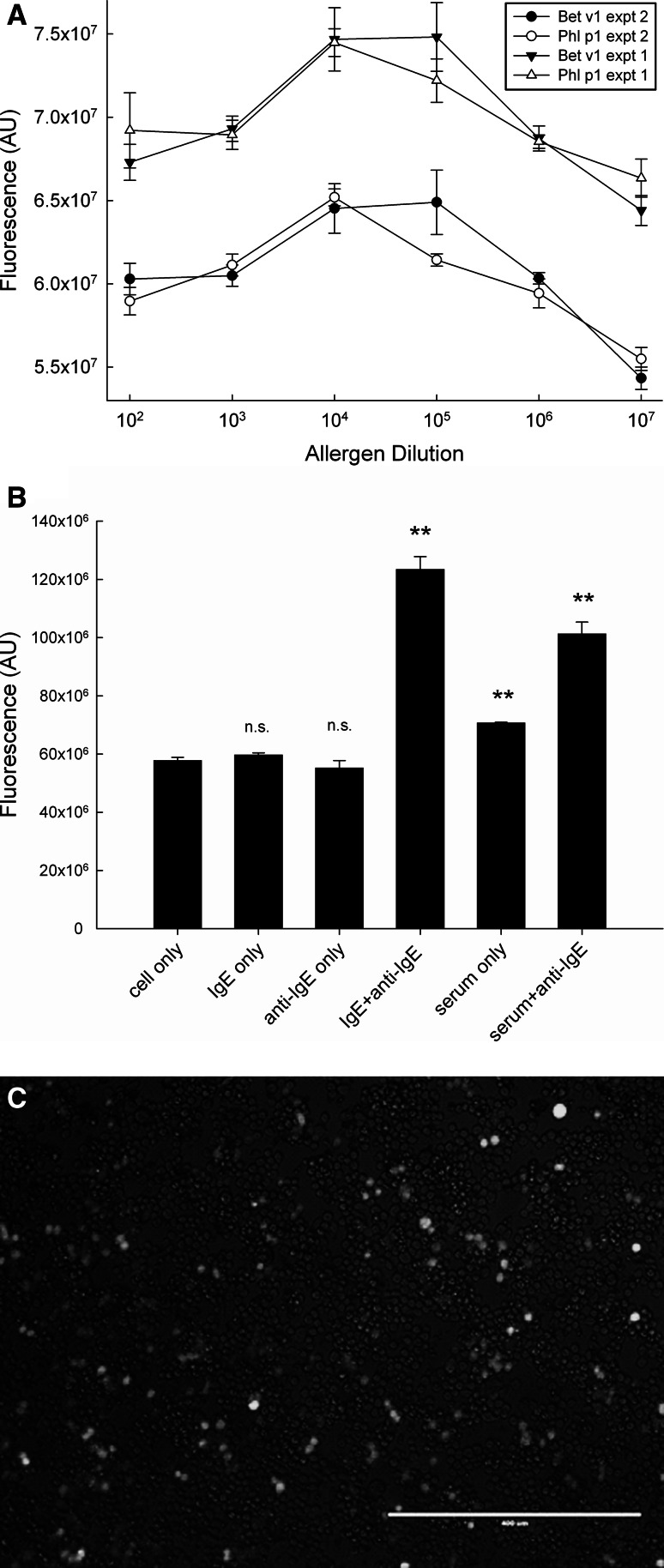
**a** Dose-dependent induction of NF-AT/GFP reporter. An RBL-703/21 clone sorted by FACS stably transfected with NF-AT-hrGFP was sensitised overnight with 1:40 diluted (v/v) serum of an allergic patient. FN-bound cells were stimulated with varying dilutions of Bet v1 (*black symbols*) or Phl p1 (*open symbols*) in two separate experiments (expt. 1: *triangles*; expt. 2: *circles*). Data shown are mean fluorescence ± SD from triplicate determinations. **b** Negative and positive control experiments for the experiments are shown in Fig. 6a. The mean ± SD from triplicate determinations of the RBL NF-AT-GFP reporter unsensitised and unstimulated (cell only), sensitised with IgE and unstimulated (IgE only), sensitised with IgE and stimulated with anti-IgE (IgE+anti-IgE), sensitised with serum and unstimulated (serum only) or sensitised with serum and stimulated with anti-IgE (serum+anti-IgE) are shown. **p < 0.01, paired Student’s *t* test. *n.s.* not significant. **c** Fluorescent microscope image (bar: 0.4 mm) of IgE/anti-IgE-stimulated RBL NF-AT-GFP reporter cells

For this purpose, we transfected a commercial NF-AT-hrGFP reporter into the humanised RBL-703/21 cell line. Stable transfectants were selected and underwent several rounds of flow cytometry sorting as described in the Methods section. The obtained stable transfectant cell line (RBL NF-AT/GFP) was sensitised with serum of a patient allergic to grass pollen and stimulated with several dilutions (ranging from 1:10^2^ to 1:10^9^) of a 1 mg/mL solution of Bet v1 and Phl p1 allergens (Fig. 6a). These experiments were performed in 384-well plates instead of microarrays due to the unavailability of equipment able to measure cell arrays without drying and fixation (see discussion). Cells were bound to the bottom wells coated with 20 μg/mL FN and sensitised for 16 h with Bet v1 and Phl p1 allergic patient serum diluted 1:40 (v/v). After addition of allergen, cells were incubated for a further 20 h, and fluorescence was measured.

As shown in Fig. 6a, fluorescence levels showed a dose-dependent bell-shaped curve with a broad optimum. Figure 6b illustrates the related set of control conditions, with unstimulated cells (cell only), cells sensitised with IgE but unstimulated (IgE only), unsensitised and anti-IgE-stimulated cells (anti-IgE only), IgE-sensitised and anti-IgE-stimulated cells (IgE+anti-IgE), serum-sensitised, unstimulated cells (serum only), and serum-sensitised anti-IgE-stimulated cells (serum+anti-IgE). These results show that an increase in GFP fluorescence (Fig. 6c) is only obtained when IgE-sensitised cells are activated by crosslinking agents (either allergen in Fig. 6a or anti-IgE antibodies in Fig. 6b). There was a small, but statistically significant fluorescence increase in serum treated but unstimulated cells, which may be attributed to low-level NF-AT translocation caused by monomeric IgE binding to the high-affinity receptor [21]. However, the fact that we did not find any effect of incubation with monoclonal myeloma IgE seems to exclude this explanation. As we did not find a similar increase with the DsRed reporter system (Fig. 8b), it is more likely to be caused by intrinsic differences between clones, rather than reflecting activation by monomeric IgE.

**Fig. 7:**
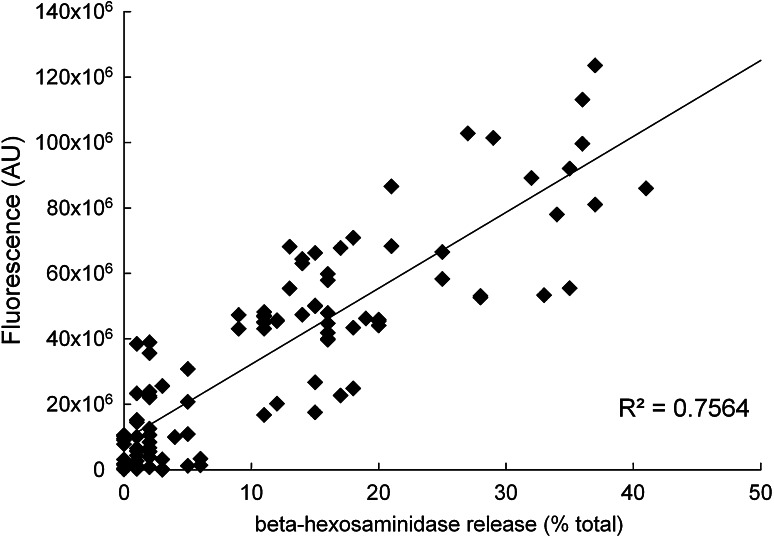
Correlation between allergen- or anti-IgE-induced GFP fluorescence and released beta-hexosaminidase activity, obtained from 102 individual experiments in which cells were sensitised with serum or IgE, and stimulated with allergen or anti-IgE, respectively

However, the data in Fig. 6b also show that there is a low signal-to-noise ratio of approximately twofold under the best activation conditions, due at least in part to the high background autofluorescence of the cells at the wavelength used for GFP measurement [22]. This raised the question whether the use of the reporter cell lines had sufficient discriminatory power in comparison with the traditional assay based on measurement of beta-hexosaminidase which is released by activated cells during degranulation. Therefore, we determined the correlation between the activation of the GFP reporter cell line and the traditional beta-hexosaminidase biochemical assay by testing in parallel using the same sera/allergen and IgE/anti-IgE-positive controls. As shown in Fig. 7, overall there was a reasonable degree of correlation (*R* ^2^ = 0.7564) with no visible trend for improved correlation in any range of activation.

**Fig. 8:**
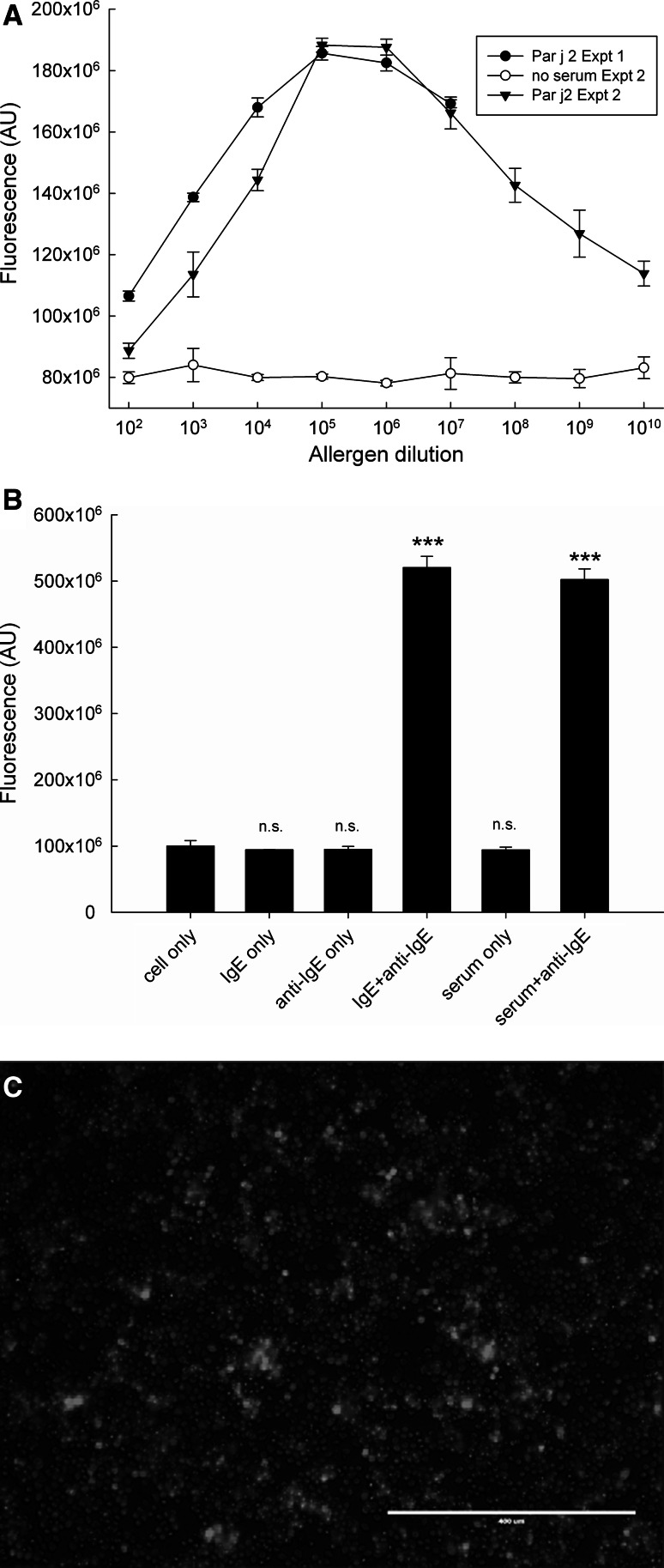
**a** Dose-dependent induction of NF-AT/DsRed reporter. An RBL-703/21 clone sorted by FACS stably transfected with NF-AT-DsRed was sensitised overnight with 1:20 (v/v) diluted pooled serum from ten allergic patients monosensitised to *Parietaria judaica*. FN-bound cells were stimulated with varying dilutions of Par j 2 (*black circles* expt.1, *black triangles* expt. 2). The graph also shows the lack of DsRed induction in cells which were not sensitised with serum in the second experiments (to control for autofluorescence of the used allergen). Data shown are mean fluorescence ± SD from triplicate determinations. **b** Negative and positive control experiments for the experiments shown in Fig. 6a. The mean ± SD from triplicate determinations of the RBL NF-AT-DsRed reporter unsensitised and unstimulated (cell only), sensitised with IgE and unstimulated (IgE only), sensitised with IgE and stimulated with anti-IgE (IgE+anti-IgE), sensitised with serum and unstimulated (serum only) or sensitised with serum and stimulated with anti-IgE (serum+anti-IgE) are shown. ***p < 0.001, paired Student’s *t* test. *n.s.* not significant. **c** Fluorescent microscope image (bar: 0.4 mm) of IgE/anti-IgE-stimulated RBL NF-AT-DsRed reporter cells

In order to improve the unacceptably low signal-to-noise ratio and fluorescence intensity of GFP proteins in this system, a new reporter system was constructed by linking the NF-AT promoter sequence with a DsRed reporter gene. Positive and stable transfectants were selected using blasticidin followed by several rounds of FACS selection as described in the Methods section. As demonstrated in Fig. 8, the use of DsRed as reporter fluorescent protein instead of GFP improved the signal-to-noise ratio, which was approximately fivefold for the positive controls (monoclonal IgE-sensitised anti-IgE-stimulated or serum-sensitised, anti-IgE-stimulated) and threefold for the experimental samples at optimum allergen concentration (see Fig. 8b).

Overall fluorescence intensity was also increased (Fig 8c). However, the main background due to the used 384-well plastics could not be eliminated or further reduced. In comparison with the use of the GFP reporter, the DsRed reporter showed a sharper, more pronounced bell-shaped activation curve upon stimulation with a serial dilution of the allergen. As can be seen from Fig. 8a, reproducibility between experiments was also good.

## Discussion

Previous work with mast cells and basophils has indicated that FcεRI surface expression is stabilised and increased up to ~17-fold after overnight incubation with its ligand IgE [23, 24]. Significant reduction of surface IgE receptor turnover has also been shown to occur in RBL cells incubated with IgE [25, 26]. However, no previous work has described the kinetics of FcεRI α-chain surface expression in humanised RBL, which is driven by a strong constitutive (e.g. CMV) promoter. Thus our first step in optimising the conditions for the basophil array was to determine the effect of incubation with IgE on surface IgE receptor expression and on the ability to immobilise IgE-sensitised RBL cells on the array surface via an anti-IgE antibody. mRNA levels for human FcεRI α chain were only modestly increased with a ~1.7-fold increase peaking after 4 h (data not shown). This suggests that the increased surface levels of FcεRI α chain observed in Fig. 1, which peaked after 8–10 h, were mainly due to a stabilisation of the receptor at the protein level rather than from induced changes in mRNA expression, in line with the findings of Furuichi et al. [25].

In many cells, stress fibres emanate from distinct areas of the plasma membrane known as focal adhesions, where clusters of integrin receptors bind to ECM proteins. Focal adhesion proteins at the intracellular surface of the plasma membrane include vinculin, paxillin and talin, which are known to link F-actin with integrins [27]. Adherence to ECM-coated surfaces results in cell spreading, reorganization of cytoskeletal F-actin and the formation of surface ruffles [14]. It was reported that treatment of RBL-2H3 cells with Ca^2+^ ionophore A23187 or anti-human IgE leads to redistribution of vinculin to the cytoskeletal fraction [28]. Although both anti-human IgE and Ca^2+^-ionophore can lead to changes of the cell surface morphology, our adhesion experiment results showed that the surface levels of the integrin VLA-4 behave very differently between the receptor- and non-receptor-mediated activation pathways. As a result, while inclusion of FN in the array is important in reducing cell losses during the washing steps, A23187 but not anti-IgE stimulation leads to a pronounced RBL cell detachment after activation even in the presence of FN. Therefore, A23187 is clearly not a suitable positive control for our RBL array. There are several reports describing an increased attachment of mast cells [16, 29, 30] to ECM proteins after activation. In such reports, attachment of primary mast cells to ECM proteins was found to depend on pre-activation, as in the case of bone marrow-derived murine or peritoneal rat mast cells, via PMA [16, 31]. However, such pre-activation is not necessary for RBL [14, 16] or MCP-5 cells [31] which are able to attach spontaneously. Thompson et al. [29] assessed attachment of A23187 or antigen-stimulated mast cells to LN and found that approximately 20 % of mast cells attached to LA; however, they did not study the detachment of previously attached cells after stimulation. Thus the apparent discrepancies between reports in the literature describing increased attachment to ECM after activation and our own, which clearly demonstrate pronounced detachment after stimulation with A23187, are due to differences in the protocol.

Another perhaps unexpected result was the ability of CO and LA to bind the RBL cells and prevent losses during the washing step. Ra et al. [15] have found that while RBL-2H3 bound to VN and FN, they did not bind to CO and LA. However, binding of RBL-2H3 cells to type I CO and LA, although less pronounced than to FN, has been described by other authors [14, 32]. Interestingly, none of the three ECM proteins appeared to increase cell attachment after IgE-dependent activation. Cell loss of CO-attached cells after A23187 treatment was very similar to FN; however, cell loss with LA was far less pronounced. This suggests that the corresponding integrins, which partially overlap between CO and LA but not with FN, are also differentially regulated by A23187 activation in RBL cells.

Regarding the use of the newly developed reporter systems, both GFP and DsRed reporter cell lines showed the expected bell-shaped dose-dependent response. A longer incubation of the reporter cells is needed in comparison with the NF-AT–Luciferase system, which is described as optimal after 3 h [20]. While we were able to detect increases in fluorescence as early as 8-h post stimulation, these changes increased steadily for at least 20 h. We do not know whether this reflects an issue of sensitivity of detection or differences in the rate of synthesis or folding of the different reporter proteins. However, the main problem here was the low signal-to-noise ratio due to the high background, ranging from 2- to 5-fold even under best stimulation conditions. Our final aim is to employ the reporter cell lines using allergen arrays with several hundreds of recombinant allergens or food extracts in parallel as previously used for Immunoglobulin determination [32]. However, the use of conventional laser array scanners is not compatible with the use of cells growing in medium, and commercially available laser microscopes do not have the necessary width of field of view to record the patterns of cellular activation at the array level. Thus our experiments with the fluorescent reporter cell lines had to be performed using 384-well plates and a Typhoon scanner, which can be used with cells immersed in aqueous culture medium. This type of scanner does not have the necessary resolution at the cellular level and the plastic materials used in 384-well cell culture plates have unacceptably high background fluorescence for this application. We are currently developing a microscope with properties which make them more amenable to the measurement of fluorescence in aqueous environment by combining high numerical aperture optics and tiling of mirror scans to obtain a high-resolution image on a large surface.

In summary, we have defined optimal conditions for the use of humanised RBL cells in array format and shown that the inclusion of FN is essential in order to avoid cell losses during the washing steps. A23187 is an unsuitable positive control in this format due to a fast downregulation of VLA-4 and subsequent detachment of cells. Using these optimised conditions, both GFP and DsRed humanised reporter cell lines were able to detect the presence of allergen-specific IgE in the sera of patients and showed the expected bell-shaped dose–response.

Our further efforts will be geared towards implementing these optimised conditions into an automated detection system which can be used for high-throughput assessment of productive IgE–allergen interactions in array format.
